# 37319 A Technology Evaluation Framework for Rural Health Research

**DOI:** 10.1017/cts.2021.542

**Published:** 2021-03-30

**Authors:** Joshua Fehrmann

**Affiliations:** University of Minnesota

## Abstract

ABSTRACT IMPACT: As technologies emerge at an increasing pace, the product developed through this work will guide rural health experts through a repeatable method of technology evaluation and selection at a faster and more reliable pace than otherwise possible. OBJECTIVES/GOALS: New technologies are emerging at an increasing pace, which leads to the question: ‘how is one to select a specific technology for their research?’ In response, this project endeavored to develop a technology evaluation and selection framework for rural health researchers. METHODS/STUDY POPULATION: The approach selected for this project included three phases. Phase one was to gain an understanding of rural health challenges, health-related emerging technologies, and rural health resources. Phase two involved using the information from phase one to select and adapt a set of technology foresight and forecasting analysis tools to be compiled within a framework. The third phase of the project was to prototype the framework, obtain researcher feedback, and iteratively implement improvements. Recommendations for the future of the framework were also developed during the third phase. RESULTS/ANTICIPATED RESULTS: The resulting product is the ‘Rural Health: Evaluation and Selection of Technology (RHEST) Framework.’ The RHEST Framework is a guide made available to use to aid in technology selection during the development of a new rural health project. The framework guides researchers through various stages, including ideation, analysis, and decision. Technology analysis tools are introduced in each stage, with links to additional information. The guide also contains a resource catalog for quick information look-up to find data sources, funding opportunities, and expert connections. Quantitative and qualitative data captured indicate that the product would add value for rural health researchers. DISCUSSION/SIGNIFICANCE OF FINDINGS: The initial version of the RHEST framework is limited in value because it is a static document and the primary audience are researchers. The value potential could improve considerably, however, if the framework were expanded to be a dynamic resource available to rural health care providers.

